# Genome-Wide Association Mapping of Seedling Heat Tolerance in Winter Wheat

**DOI:** 10.3389/fpls.2018.01272

**Published:** 2018-09-04

**Authors:** Frank Maulana, Habtamu Ayalew, Joshua D. Anderson, Tadele T. Kumssa, Wangqi Huang, Xue-Feng Ma

**Affiliations:** ^1^Noble Research Institute, Ardmore, OK, United States; ^2^Institute of Food Crops, Yunnan Academy of Agricultural Sciences, Kunming, China

**Keywords:** wheat, GWAS, QTL, heat stress, seedling stress

## Abstract

Heat stress during the seedling stage of early-planted winter wheat (*Triticum aestivum* L.) is one of the most abiotic stresses of the crop restricting forage and grain production in the Southern Plains of the United States. To map quantitative trait loci (QTLs) and identify single-nucleotide polymorphism (SNP) markers associated with seedling heat tolerance, a genome-wide association mapping study (GWAS) was conducted using 200 diverse representative lines of the hard red winter wheat association mapping panel, which was established by the Triticeae Coordinated Agricultural Project (TCAP) and genotyped with the wheat iSelect 90K SNP array. The plants were initially planted under optimal temperature conditions in two growth chambers. At the three-leaf stage, one chamber was set to 40/35°C day/night as heat stress treatment, while the other chamber was kept at optimal temperature (25/20°C day/night) as control for 14 days. Data were collected on leaf chlorophyll content, shoot length, number of leaves per seedling, and seedling recovery after removal of heat stress treatment. Phenotypic variability for seedling heat tolerance among wheat lines was observed in this study. Using the mixed linear model (MLM), we detected multiple significant QTLs for seedling heat tolerance on different chromosomes. Some of the QTLs were detected on chromosomes that were previously reported to harbor QTLs for heat tolerance during the flowering stage of wheat. These results suggest that some heat tolerance QTLs are effective from the seedling to reproductive stages in wheat. However, new QTLs that have never been reported at the reproductive stage were found responding to seedling heat stress in the present study. Candidate gene analysis revealed high sequence similarities of some significant loci with candidate genes involved in plant stress responses including heat, drought, and salt stress. This study provides valuable information about the genetic basis of seedling heat tolerance in wheat. To the best of our knowledge, this is the first GWAS to map QTLs associated with seedling heat tolerance targeting early planting of dual-purpose winter wheat. The SNP markers identified in this study will be used for marker-assisted selection (MAS) of seedling heat tolerance during dual-purpose wheat breeding.

## Introduction

Wheat (*Triticum aestivum* L.) is one of the most important feed and food crops in the world and it covers more cultivable land globally than any other crop. Moreover, it provides food for 36% of the world’s population (Cossani and Reynolds, 2012; Prerna et al., 2013; Kim and Anderson, 2015). In the southern Great Plains of the United States, including Oklahoma and Texas, dual-purpose wheat grown for cool season grazing is seeded at least 2–3 weeks earlier than wheat grown for grain only to increase fall to winter forage production. However, early planting in the fall often coincides with high temperatures that affect seed germination, seedling growth, and development, eventually resulting in reduced forage and grain yield. As the global climate continues to change, the severity and frequency of high temperature stress is likely to increase, thereby resulting in reduction of productivity of important crops including wheat. Climate predictions show that, by the end of the 21st century, the average global temperature is expected to increase by 1–4°C (Driedonks et al., 2016). Therefore, development of dual-purpose wheat cultivars with tolerance to heat stress during the seedling stage is crucial for early planting in the region.

Several studies have outlined the effects of heat stress on plant morphological, physiological, and biochemical processes at various growth stages of wheat (Cossani and Reynolds, 2012; Paliwal et al., 2012; Feng et al., 2014; Chaturvedi et al., 2017). The effects of heat stress during the seedling stage include reduction of photosynthesis, chlorophyll content, respiration rate, and death of the seedlings due to excessive dehydration of leaves beyond the permanent wilting point (Ristic et al., 2007; Cossani and Reynolds, 2012). Research findings in the past indicated that heat stress causes swelling of the thylakoid membrane and malfunction of photosystem II involved in photosynthetic activity (Ristic et al., 2007; Talukder et al., 2014). Chlorophyll is harbored in the thylakoid membrane and when this membrane is damaged by the stress, chlorophyll content is reduced (Ristic et al., 2008). However, phenotypic variability for heat tolerance among genotypes has been studied during the reproductive stage but limited information is available for the seedling stage.

High temperature stress at the grain filling stage has been reported to reduce the yield and quality of wheat (Wardlaw et al., 2002; Schapendonk et al., 2007; Stratonovitch and Semenov, 2015), sorghum [*Sorghum bicolor* (L.) Moench] (Prasad et al., 2008), maize (*Zea mays* L.) (Yang et al., 2015), and rice (*Oryza sativa* L.) (Shi et al., 2016). Heat stress has been found to reduce wheat yield by 33.6% and by more than 50% (Chatrath et al., 2007; Joshi et al., 2007). The yield potential of wheat is rarely attained, particularly when moderate heat stress occurs and alternates with periodic extreme heat stress (Mason et al., 2011). Heat tolerance is a polygenic trait that is controlled by many genes with minor effects on the phenotype. Therefore, selection of heat stress tolerance under field conditions is very challenging because of its genetic complexity, weather variability and the influence of genotype-by-environment interaction effect. In this regard, identification of QTLs and molecular markers associated with tolerance to heat stress is crucial for improving breeding efficiency using marker-assisted selection (MAS).

To date, dissection of QTLs for heat tolerance in wheat has been mainly conducted during the grain filling stage using bi-parental mapping populations (Mason et al., 2010; Vijayalakshmi et al., 2010; Talukder et al., 2014). These studies identified various major and minor QTLs for vegetative and reproductive stage traits on different wheat chromosomes. For example, five QTLs for heat tolerance in wheat were detected on chromosomes 1B, 1D, 2B, 6A, and 7A (Talukder et al., 2014). Similarly, two QTLs for heat tolerance have been detected on chromosomes 2B and 5B in a spring wheat mapping population (Butler, 2002). Again in other studies, QTLs for heat tolerance during the grain filling stage have been found on several chromosomal regions including 1A, 1B, 2B, 3B, 5A, and 6D (Mason et al., 2010). In addition, QTLs associated with yield components and physiological traits, such as stay green and senescence of wheat, were found on chromosomes 2A, 3A, 4A, 6A, 6B, and 7A (Vijayalakshmi et al., 2010).

Moreover, using a meta-analysis strategy, major QTLs associated with heat tolerance were detected on chromosomes 1B, 2B, 2D, 4A, 4D, 5A, and 7A (Acuña-Galindo et al., 2015). Similarly, another significant locus on chromosome 3B, associated with the heat susceptibility index of yield components, was identified using a bi-parental mapping population (Mason et al., 2010). Although linkage mapping using bi-parental mapping populations has successfully identified heat tolerance QTLs, it requires a large amount of resources and time to develop mapping populations such as recombinant inbred lines (RILs). In addition, it relies on recent recombination resulting in low mapping resolution and only alleles differing in the parents are considered with this approach. On the other hand, with GWAS, diverse individuals are used without developing new mapping populations, making it less expensive. Historic recombination events existing in the population are leveraged in GWAS, thereby resulting in high mapping resolution compared with linkage mapping. However, GWAS requires higher marker density than traditional QTL mapping because linkage disequilibrium (LD) is in general much lower in a GWAS population than in a bi-parental population.

The GWAS approach has been used to discover genes controlling both polygenic and monogenic traits. For example, QTLs associated with important traits such as disease resistance (Tadesse et al., 2015; Arruda et al., 2016; Liu et al., 2017), yield, and grain quality traits (Tadesse et al., 2015) in wheat have been discovered with GWAS. QTLs associated with heat tolerance in wheat have also been detected using GWAS (Mondal et al., 2015).

Although heat tolerance during the reproductive stage of wheat has been well characterized, heat stress during the seedling stage is not studied. Therefore, the objectives of this study were: (1) to map QTLs associated with seedling heat tolerance in wheat and (2) to identify SNP markers for MAS of seedling heat tolerance during dual-purpose wheat breeding in the southern Great Plains of the United States.

## Materials and Methods

### Genetic Materials and Phenotyping

A set of 200 lines, selected based on genetic diversities from a hard red winter wheat association mapping panel consisting of 299 wheat lines from the Triticeae Coordinated Agricultural Project (TCAP^1^), was used in this study. The association mapping panel is composed of representative winter wheat lines across the Great Plains (Grogan et al., 2016). The experiments were conducted in two growth chambers. A high-temperature treatment (40/35°C day/night) to mimic heat stress was induced at the three-leaf stage for 14 days in one chamber, and an optimal-temperature treatment (25/20°C day/night) was used as a control in the other chamber. Photoperiod and light intensity in both growth chambers were set at 16 h and 400 μml m^-2^ s^-1^, respectively. The plants were planted in 72-well flat trays in a randomized complete block design with three biological replicates of each line. The trays were randomly arranged and periodically moved around to avoid positional effect. Throughout the experimentation, plants were watered as needed in both growth chambers to ensure no drought stress.

Data were collected on leaf chlorophyll content, shoot length, number of leaves per seedling, and seedling recovery. Leaf chlorophyll content was measured using a self-calibrating SPAD chlorophyll meter (Model 502, Spectrum Technologies, Plainfield, IL, United States). Three measurements of leaf chlorophyll content were taken per line, and the average was used for statistical analysis. Shoot length was measured from the soil surface to the tip of the longest leaf. Leaf chlorophyll content and shoot length were measured 10 days after heat stress treatment. Number of leaves per seedling was recorded as the average number of leaves counted from three seedlings, 14 days after the seedlings were exposed to heat stress. Seedling recovery was the percentage of seedlings that were able to recover 7 days after removal of heat stress treatment. Heat stress response, referred to as trait relative difference (TRD), was calculated as the difference between trait performance at optimal and high temperatures, and then divided by performance at optimal temperature. The experiment was repeated six times (i.e., six runs) using the same two chambers.

### Phenotypic Data Analysis

Analysis of variance of the phenotypic data was performed using the Statistical Analysis System (SAS) software V9.3 (SAS Institute, 2011) to assess the effects of genotype, run, and genotype-by-run interaction. All sources of variation were considered as random effects. All other variances besides genotype and experimental run were pooled as residuals.

### SNP Genotyping

The wheat lines were genotyped using the wheat iSelect 90K SNP genotyping array (Wang et al., 2014; Guttieri et al., 2017), which generated 21,555 SNPs. After SNPs with minor allele frequency (MAF) of less than 5% and missing data of more than 10% were filtered out, a total of 15,574 SNPs remained and were used for analysis. The genetic positions of the SNP markers used in this study were based on the consensus map developed using eight wheat mapping populations (Wang et al., 2014).

### Population Structure, Kinship, and Linkage Disequilibrium Analyses

The genetic structure of the panel was assessed using the STRUCTURE program, principal component analysis (PCA), and neighbor-joining (NJ) tree analysis. The STRUCTURE program version 2.3.4 (Falush et al., 2003) was used to estimate the number of groups (*K*) and the membership coefficients. A model-based Bayesian clustering approach was performed, where the number of assumed groups was set from *k* = 1 to 10. During STRUCTURE analysis, a Markov chain Monte Carlo (MCMC) of 15,000 burn-in replicates followed by 15,000 iterations was run and repeated five times using an admixture model. Due to lots of admixtures in the panel, the STRUCTURE results were verified by comparing the results to other analyses. The optimal number of groups in this panel was determined based on the point where the posterior probability [LnP(D)] began to plateau from the STRUCTURE analysis (Casa et al., 2008) and the NJ tree analysis. The principal components (PCs) were calculated using the R function *princomp*, while the NJ tree analysis was performed in TASSEL version 5.2.28 (Bradbury et al., 2007). To determine the number of PCs to use in clustering and GWAS analysis, a scree plot was generated by plotting the percentage of variances explained by the first 10 PCs against the number of PCs. Based on this, the optimal number of PCs (where the “*elbow*” point occurred) was selected. The analysis of K between lines was performed following the identity-by-state method (Endelman and Jannink, 2012).

Linkage disequilibrium among pairs of SNP markers was performed with the TASSEL software using 3,484 tag SNPs selected using the R package SNPRelate (Zheng, 2013). LD for within and across the three wheat genomes (A, B, and D) was estimated as a squared allele frequency correlation (*r^2^*) between SNP marker pairs. All SNP marker pairs with *p*-values of less than 0.001 were considered to be in significant LD. LD decay distance was estimated by plotting the scatterplot of LD *r^2^* values between marker pairs and the genetic distance (in cM) using the R package SNPRelate (Zheng, 2013), while the trend line was fitted by second-degree LOESS (Cleveland, 1979). To determine whether significant SNP markers associated with the trait on each chromosome were in LD with the highest -log_10_(*p*-value) SNP hit, LD analysis was performed on every chromosome where significant QTLs were detected.

### Genome-Wide Association Mapping Analysis

Genome-wide association mapping was performed with the Genome Association and Prediction Integrated Tool (GAPIT) (Lipka et al., 2012). For the Q model, three PCs that were selected based on scree plot generated from PCA were included in the model as fixed-effect covariate (Zhao et al., 2007) to correct for population structure. In the K model, the K matrix between individuals was calculated and included in the model as random-effect covariate. For mixed linear model (MLM), both the population structure (PCs) and K matrix were included in the model as fixed and random-effect covariates, respectively (Yu et al., 2006).

For the Q model, the following equation was used:

Y = Xβ + e

*Y* is the vector of phenotypic values, *X* is the design matrix, β is the vector consisting of SNP markers and population structure (PCs) included in the model as fixed effects, and *e* is the random error.

For the K and MLM models, the following equation was used:

Y = Xβ + Zμ + e,

where *Z* is the design matrix and μ is the vector comprising additive genetic effects considered as random. In the K-model, β contains only markers and μ contains the K-matrix, while in the MLM, β has both markers and population structure (PCs), and μ has the K matrix. Significant QTLs were initially tested based on a false discovery rate (FDR)-adjusted *p*-value of 0.05 following a step-wise procedure (Benjamini and Hochberg, 1995), which is very stringent (Müller et al., 2011). However, a lower threshold, unadjusted significance *p-*value <0.001, was eventually used to declare significance since the FDR is too stringent in the current study. Visualization of the significant QTLs and SNPs was done using Manhattan plots, generated using the R package *qqman* (Turner, 2018).

### Candidate Gene Analysis

A BLAST search was performed against the newly released wheat reference sequence hosted by the URGI-INRA^2^ and the National Center for Biotechnology Information (NCBI) database to identify candidate genes or related proteins with DNA sequences similar to the SNPs significantly associated with seedling heat tolerance-related traits detected in this study.

## Results

### Phenotypic Data Analysis

**FIGURE 1:**
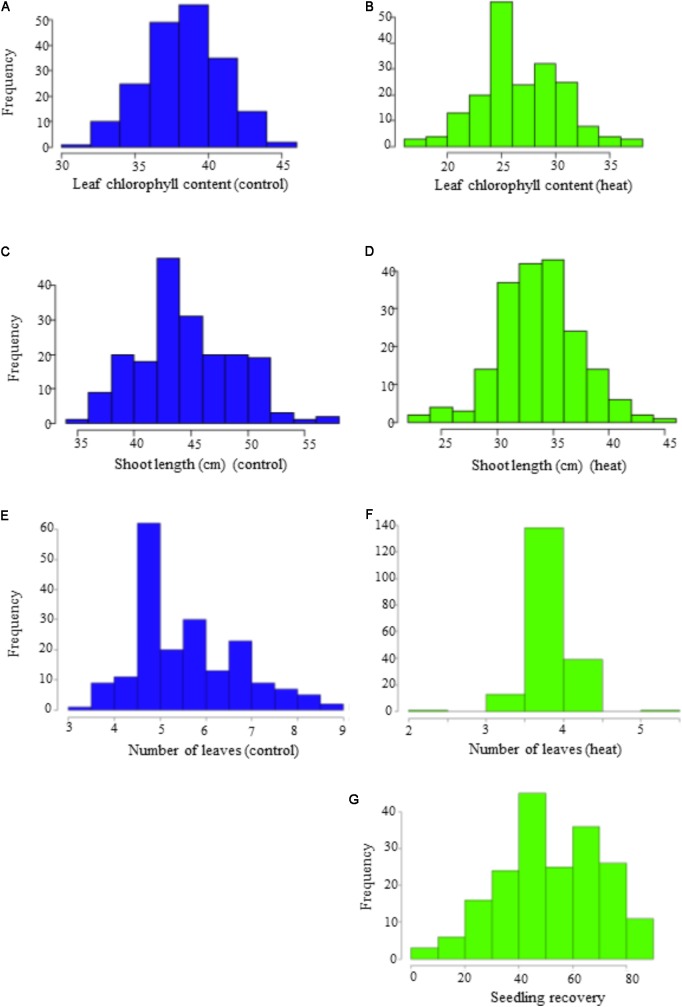
Frequency distribution of the seedling traits observed at optimal (OT) and heat-stressed (HS) growth conditions in the panel. **(A,B)** Leaf chlorophyll content at optimum and heat-stressed growth conditions; **(C,D)** shoot length (cm) at optimum and heat-stressed growth conditions; **(E,F)** number of leaves at optimum and heat-stressed growth conditions; **(G)** seedling recovery after removal of the heat stress.

Phenotypic variation was observed among genotypes for all traits in both temperature regimes (**Table 1**). Frequency distribution of the lines for the investigated traits at optimal and heat-stressed growth conditions are presented in **Figure 1**. Mean leaf chlorophyll content at optimal temperature was 38.3 with a range from 31.8 to 44.9, while for heat-stressed plants, mean leaf chlorophyll content was 26.7, ranging from 17.0 to 37.1. At optimal temperature, mean shoot length was 44.9 cm, ranging from 35.0 to 56.5 cm, whereas at heat-stressed growth condition, the mean value was 33.8 cm, and the range was from 23.5 to 44.4 cm. Mean number of leaves per seedling was six at optimal temperature compared with four at heat-stressed growth condition. For the number of leaves per seedling, phenotypic variation among lines was very small as shown in **Figure 1** because almost all plants were at three-leaf stage when the experiment started. As a result, variation in number of leaves per seedling among lines was very small by the end of 14-day temperature treatment. As for seedling recovery, on average, 52.3% of seedlings were able to recover after the removal of heat stress treatment (**Table 1**). Overall, heat stress reduced leaf chlorophyll content, shoot length and number of leaves per seedling by 30.3, 25.0, and 32.2%, respectively.

**Table 1:** Seedling trait performance of the winter wheat lines under optimal and high temperatures in the present study.

Trait	Optimal temperature (25/20°C)			Heat stress (40/35°C)		
		
	Mean	Range	SD	Mean	Range	SD
Leaf chlorophyll content (SPAD)	38.30	31.82–44.89	2.60	26.70	17.03–37.14	3.85
Shoot length (cm)	44.87	35.00–56.50	4.28	33.83	23.50–44.38	3.64
Number of leaves per seedling	6	3–9	1.14	4	3–5	0.25
Seedling recovery (%)	N/A	N/A	N/A	52.32	6.25–89.59	18.48


*SD, standard deviation; N/A, not applicable.*

### Population Structure Analysis

**FIGURE 2:**
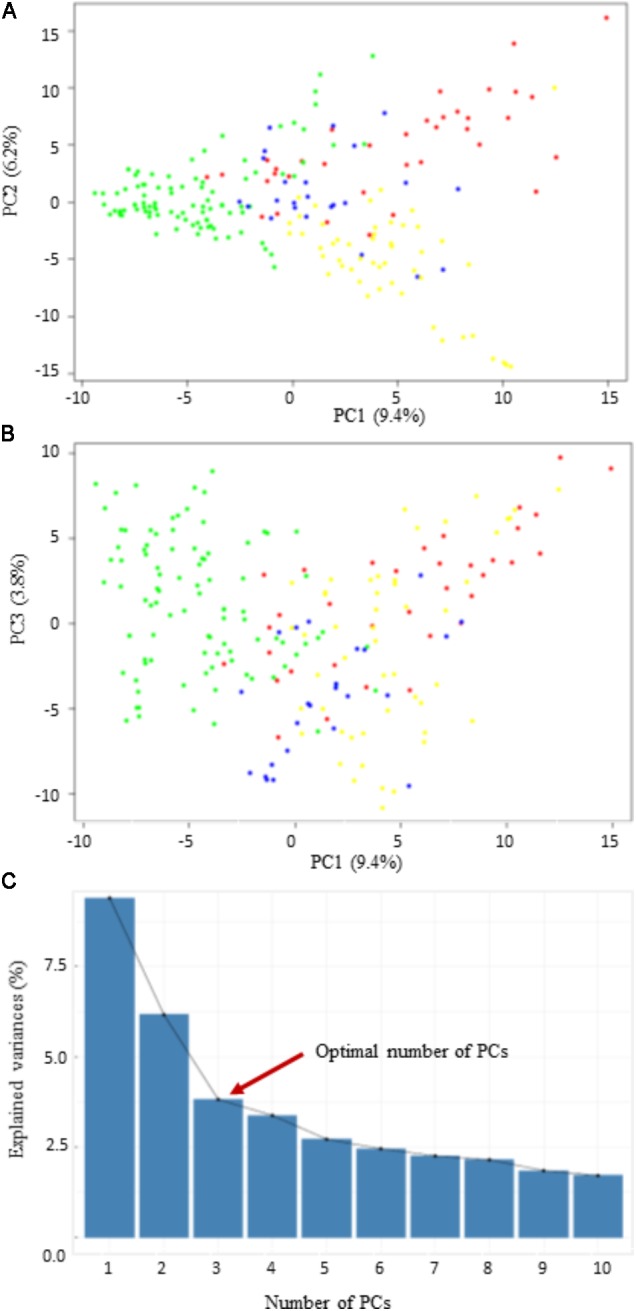
Scatter plots of the first three principal components (PCs). **(A,B)** The distribution of the 200 winter wheat lines in PC1 vs. PC2, and PC1 vs. PC3; **(C)** the scree plot shows the variance explained by the first 10 PCs.

Three different clustering methods, PCA, NJ tree analysis, and STRUCTURE analysis, were compared to assess their agreement in the pattern of structuring of this panel. PCA divided the panel into four main groups with lots of admixture (**Figure 2**). However, the PCA revealed that the population structure in this panel is very low since the first three PCs collectively explained only about 19.4% of the total variance. The first principal component (PC1) explained about 9.4%, while the second (PC2) and the third (PC3) explained about 6.2 and 3.8% of the total variance, respectively (**Figure 2**). According to the NJ tree analysis, this panel can also be divided into four major groups (G1, G2, G3, and G4), based mainly on geographic origins and pedigree information (**Supplementary Figure S1A**). For example, in the first main group (G1), majority of the lines were from the Oklahoma State University and the Texas A&M University. Most lines with a common parent in their pedigree tended to cluster into the same group. For example, the majority of the lines assigned to G1 had “Jagger” as one of the parents in their pedigree. The largest number of lines forming G2 originated from the University of Nebraska breeding program, followed by the Kansas State University and the Colorado State University. Group G3 was dominated by wheat lines from the AgriPro Syngenta followed by those from the University of Nebraska wheat breeding program. Finally, the largest number of lines in G4 came from the Texas A&M University, followed by those from the Oklahoma State University.

The STRUCTURE program also stratified panel into four groups but with a lot of admixtures (**Supplementary Figure S1B**). The lack of a distinct clustering pattern observed in this panel is because there is a high degree of relatedness among lines included in this study due to sharing genetic materials among wheat breeding programs. For GWAS analysis, we used the three PCs from the PCA as a fixed-effect covariate in the Q and MLM to correct for population structure.

### Linkage Disequilibrium Analysis

After filtering using the R package SNPRelate (Zheng, 2013), 3,484 tag SNPs were obtained for LD analysis. The majority of SNP markers were distributed across the wheat A and B genomes with 41% (1439 SNPs) and 45% (1568 SNPs), respectively, while the D genome had the lowest number (343) of SNPs (10%). In addition, the B genome had the highest number of SNP markers per cM (7.19), seconded by A genome (5.97) and D genome (1.53). On A genome, 29.5% of SNP marker pairs were in significant LD (*p* < 0.001), while on B and D genomes, 32.8 and 14.0% of SNP marker pairs were in significant LD (**Supplementary Table S1**). The scatter plots of the allele frequency correlations (*r*^2^) between the SNP marker pairs and the genetic distance (in cM) within each of the three wheat genomes (A, B, and D) are presented in **Supplementary Figure S2**. The data showed that LD decayed to <0.1 at 9.7 cM in A genome, 9.8 cM in B genome, and 10.9 cM in D genome.

### Genome-Wide Association Mapping Analysis

**FIGURE 3:**
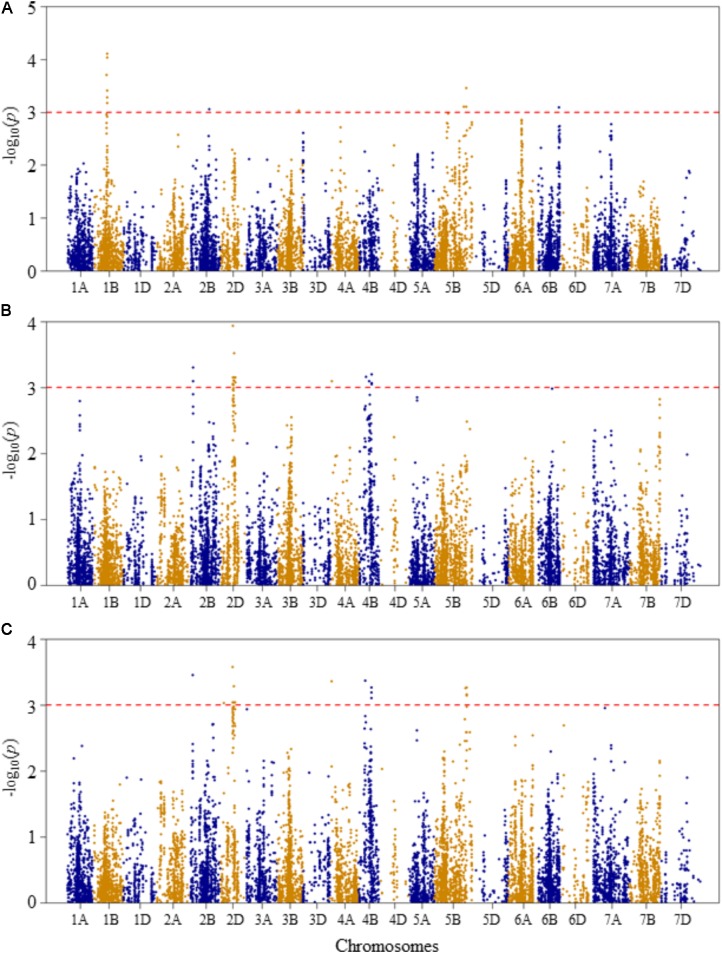
Manhattan plots of GWAS conducted on leaf chlorophyll content of the association mapping panel. **(A)** Optimal temperature; **(B)** heat stressed temperature; and **(C)** heat stress response using the trait relative difference between the two temperature treatments.

**FIGURE 4:**
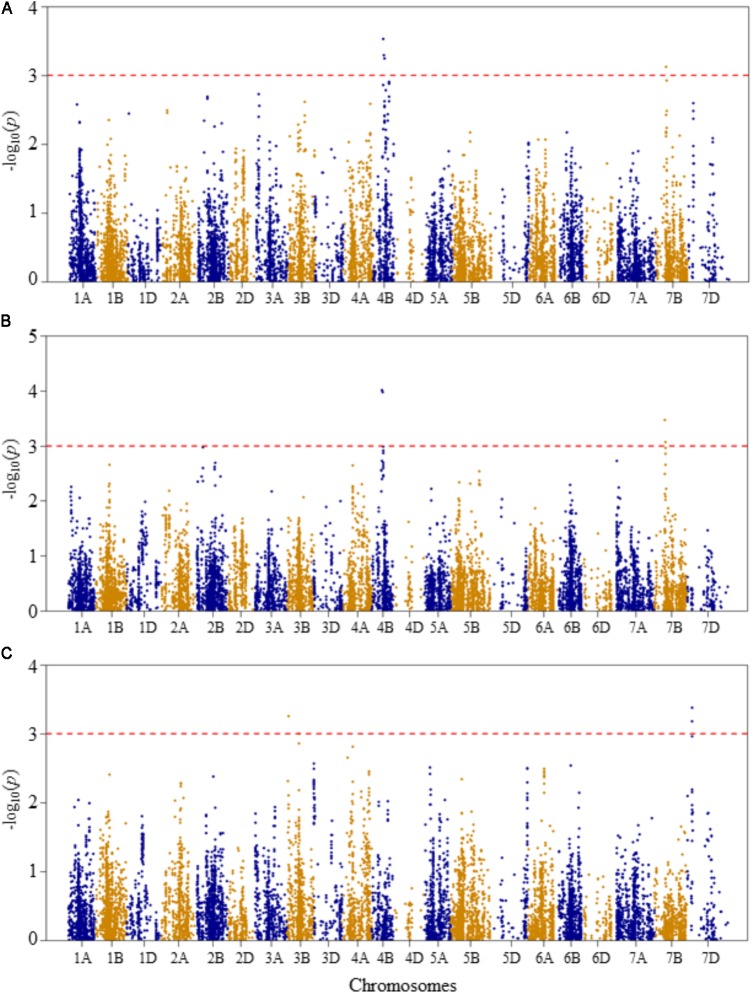
Manhattan plots of GWAS conducted on shoot length of the association mapping panel. **(A)** Optimal temperature; **(B)** heat stressed temperature; and **(C)** heat stress response using the trait relative difference between the two temperature treatments.

**FIGURE 5:**
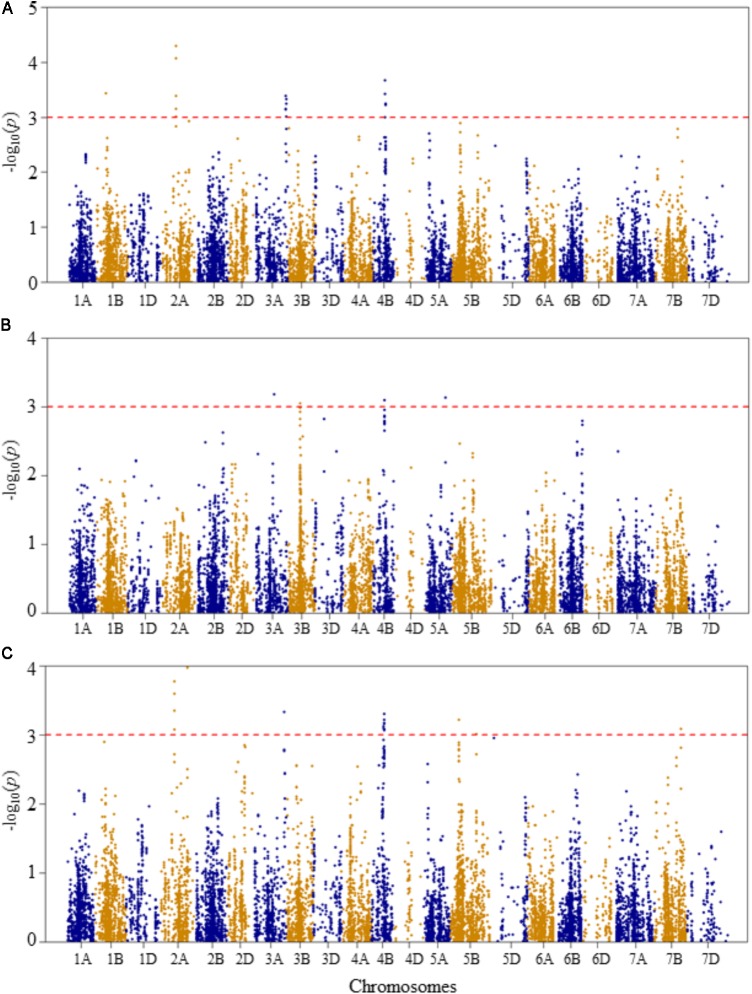
Manhattan plots of GWAS conducted on number of leaves per seedling of the association mapping panel. **(A)** Optimal temperature; **(B)** heat stressed temperature; and **(C)** heat stress response using the trait relative difference between the two temperature treatments.

**FIGURE 6:**
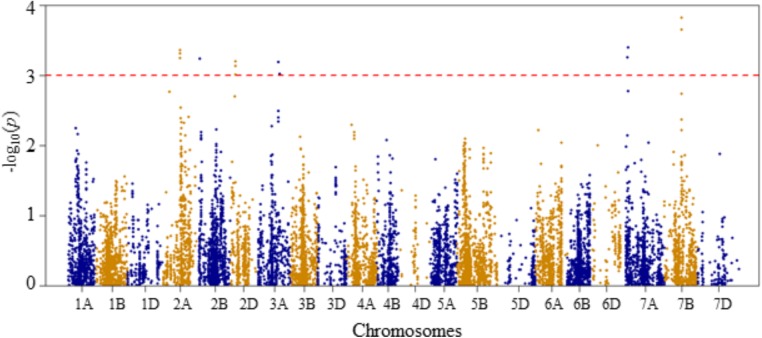
Manhattan plot of GWAS conducted on seedling recovery after removal of heat stress treatment of the association mapping panel.

Compared to Q and K models, MLM has high statistical power for controlling false positives. Therefore, in this study MLM was chosen as the appropriate model for reporting QTL mapping results. The quantile–quantile (Q–Q) plots of *p*-values comparing the uniform distribution of the expected –log_10_(*p*) to the observed –log_10_(*p*) of all evaluated traits are presented as **Supplementary Figure S3**. Genome-wide association mapping analysis results for all traits using the MLM are presented in **Figures 3–6**. The QTLs and the SNP markers significantly associated with seedling traits at optimal and heat-stressed growth conditions, as well as heat stress responses of all traits are presented in **Supplementary Table S2**. Although, no QTLs were declared significant at a FDR of 0.05, some SNPs were significant at unadjusted significance *p-*value <0.001 at optimal and/or heat-stressed growth conditions.

For leaf chlorophyll content at the optimal temperature, five QTLs, represented by 15 SNPs, were detected significant based on unadjusted significance *p-*value <0.001 in chromosomes 1B, 2B, 3B, 5B, and 6B (**Figure 3A** and **Supplementary Table S2**). The first QTL (*QLCCOT.nri-1B*) region was represented by six SNPs, which were mapped within genetic distance of 78–82 cM on chromosome 1B, and together accounted for 42.9% of the total phenotypic variation in leaf chlorophyll content at the optimal temperature. The second QTL region (*QLCCOT.nri-2B*), represented by four SNPs, was mapped at the genetic position of 119 cM on chromosome 2B. The four markers together explained 23.3% of the phenotypic variation in leaf chlorophyll content. On chromosome 5B, one QTL (*QLCCOT.nri-5B*) was mapped at 171–184 cM, which explained 18.6% of the phenotypic variation. On chromosomes 3B and 6B, two QTLs, *QLCCOT.nri-3B* (124 cM) and *QLCCOT.nri-6B* (121 cM) were detected collectively accounted for 11.7% of the phenotypic variation. Overall, the most significant SNPs for the trait were IWB9175 (80 cM), IWB14950 (80 cM), and IWB27292 (78 cM) on chromosome 1B, which collectively explained about 23.8% of the total phenotypic variation in leaf chlorophyll content under optimum growth temperature.

For leaf chlorophyll content at the heat-stressed growth condition, six QTLs were detected on chromosomes 2B, 2D, 4A, and 4B (**Figure 3B** and **Supplementary Table S2**). The first QTL (*QLCCHS.nri-2B*) was located on chromosome 2B, and explained 12.1% of the phenotypic variation of the trait. On chromosome 2D, one QTL, *QLCCHS.nri-2D* (71–86 cM) was detected. This QTL was represented by 37 SNPs, explaining phenotypic variation in leaf chlorophyll content at heat-stressed growth condition ranging from 5.7 to 7.8%. On chromosome 4A, one QTL (*QLCCHS.nri-4A*) was found and mapped at 9 cM. This QTL explained about 5.8% of the phenotypic variation. In addition, three QTLs (*QLCCHS.nri-4B.1, QLCCHS.nri-4B.2*, and *QLCCHS.nri-4B.3*) were detected at 42, 60, and 76 cM on chromosome 4B, respectively. The phenotypic variation explained by these QTLs ranged from 5.8 to 17.5%. The most significant SNP markers associated with leaf chlorophyll content under heat-stressed growth condition were IWB28109 (71 cM) and IWB65632 (77 cM) on chromosome 2D, and IWB55435 (27 cM) on chromosome 2B (**Supplementary Table S2**). These three SNP markers together accounted for 21.4% of the phenotypic variation of leaf chlorophyll content at heat-stressed growth condition.

For heat stress response of the leaf chlorophyll content, i.e., the relative difference under the two growth temperatures, seven QTLs were identified on chromosomes 2B, 2D, 4A, 4B, and 5B (**Figure 3C** and **Supplementary Table S2**). The QTLs were represented by 39 SNPs significantly associated with heat stress response. A single QTL (*QLCCHR.nri-2B*) was detected on chromosome 2B, and it was mapped at a genetic position of 27 cM. The phenotypic variation explained by this QTL was 6.8%. On chromosome 2D, two QTLs: *QLCCHS.nri-2D.1* (22 cM) and *QLCCHS.nri-2D.2* (71–85 cM) were identified. The two QTLs on 2D were represented by 29 SNPs, which accounted for 5.8–7.1% of the total phenotypic variation in heat stress response of leaf chlorophyll content. Furthermore, one QTL was mapped at 9 cM on chromosome 4A, and it accounted for 6.6% of the phenotypic variation in heat stress response of leaf chlorophyll content. On chromosome 4B, two QTLs (*QLCCHR.nri-4B.1* and *QLCCHR.nri-4B.2*) were detected at genetic positions of 40 and 76 cM, respectively. Similarly, on chromosome 5B, one QTL (*QLCCHR.nri-5B*) was mapped at 182–189 cM. The QTL on 5B was represented by four SNPs which together explained about 25.1% of total phenotypic variation in heat stress response of the trait (**Figure 3C** and **Supplementary Table S2**). The most significant SNPs were IWB28109 at 71 cM on 2D, IWB55435 at 27 cM on 2B and IWB48055 at 40 cM on 4B. These SNP markers accounted for 6.6–7.1% of the phenotypic variation in heat stress response of leaf chlorophyll content.

Overall, the data suggest that the leaf chlorophyll content QTLs associated with heat stress or heat response are located on chromosomes 2B, 2D, 4A, 4B, and 5B based on the QTLs detected for heat response of the trait, or the QTLs detected under heat-stressed but not under the optimum condition (**Table 2**).

**Table 2:** Heat stress responding QTL at the seedling stage of wheat in the present study.

Trait	Chr	Position (cM)	QTL for heat stress only^1^		QTL for heat response^2^	
				
			QTL Name	*R*^2^ (%)	QTL Name	*R*^2^ (%)
Leaf chlorophyll content	2B	27.2	QLCCHS.nri-2B	12.13	QLCCHR.nri-2B	6.80
Leaf chlorophyll content	2D	22.46			QLCCHR.nri-2D.1	5.80
Leaf chlorophyll content	2D	70.65–85.97	QLCCHS.nri-2D	24.64	QLCCHR.nri-2D.2	18.78
Leaf chlorophyll content	4A	8.61	QLCCHS.nri-4A	5.82	QLCCHR.nri-4A	6.58
Leaf chlorophyll content	4B	39.93–41.65	QLCCHS.nri-4B.1	5.96	QLCCHR.nri-4B.1	6.59
Leaf chlorophyll content	4B	59.94	QLCCHS.nri-4B.2	5.83		
Leaf chlorophyll content	4B	75.65	QLCCHS.nri-4B.3	17.53	QLCCHR.nri-4B.2	18.51
Leaf chlorophyll content	5B	182.15–188.58			QLCCHR.nri-5B	24.85
Shoot length (cm)	3B	9.7			QSLHR.nri-3B.1	6.21
Shoot length (cm)	3B	67.17			QSLHR.nri-3B.2	5.64
Shoot length (cm)	7D	26.92			QSLHR.nri-7D	12.55
Shoot length (cm)	2A	150.11			QLNHR.nri-2A.2	8.29
Number of leaves per seedling	3A	111.62	QLNHS.nri-3A	6.22		
Number of leaves per seedling	3A	177.24			QLNHR.nri-3A	6.71
Number of leaves per seedling	3B	65.72	QLNHS.nri-3B	5.91		
Number of leaves per seedling	4B	68.45–71.46			QLNHR.nri-4B	13.06
Number of leaves per seedling	5A	114.97	QLNHS.nri-5A	6.12		
Number of leaves per seedling	5B	49.02			QLNHR.nri-5B.1	12.88
Number of leaves per seedling	5B	144.26			QLNHR.nri-5B.2	5.93
Number of leaves per seedling	7B	145.29			QLNHR.nri-7B	6.11
Seedling recovery (%)	2A	95.75	QSRHS.nri-2A	32.98	N/A	
Seedling recovery (%)	2B	19.16	QSRHS.nri-2B	6.48	N/A	
Seedling recovery (%)	2D	26.05	QSRHS.nri-2D	18.55	N/A	
Seedling recovery (%)	3A	123.05–128.87	QSRHS.nri-3A	12.33	N/A	
Seedling recovery (%)	7A	42.08–43.47	QSRHS.nri-7A	13.41	N/A	
Seedling recovery (%)	7B	89.82	QSRHS.nri-7B	23.35	N/A	


*(1) QTL detected under heat stressed temperature but not at optimum temperature; (2) QTL detected using heat response, which is the trait relative difference between the two temperature treatments; QTL were declared significant based on unadjusted *p*-value <0.001; *R*^*2*^ is phenotypic variance explained by each QTL expressed as a percentage.*

For shoot length at the optimal growth temperature, two QTLs represented by four SNPs were detected significant at unadjusted *p-*value <0.001 (**Figure 4A** and **Supplementary Table S2**). The first QTL (*QSLOT.nri-4B*), represented by three SNPs, was mapped at 57–63 cM on chromosome 4B, explaining 17.4% of the phenotypic variation of shoot length at the optimal growth temperature. The other QTL (*QSLOT.nri-7B*) was mapped at 54 cM on chromosome 7B with about 5.3% of the phenotypic variation in shoot length.

On the other hand, at heat-stressed growth condition, the same two QTLs for shoot length were also found on chromosomes 4B and 7B (**Figure 4B** and **Supplementary Table S2**). On chromosome 4B, the QTL (*QSLHS.nri-4B*) was mapped at genetic position ranging from 57 to 60 cM. This QTL explained 12.8% of the phenotypic variation in shoot length. The QTL (*QSLHS.nri-7B*) on chromosome 7B was represented by two SNPs and mapped within 54–58 cM. Together, the two SNP markers explained 10% of the phenotypic variation in shoot length at heat-stressed growth condition. The most significant markers were the same markers that were detected at optimal growth condition, located on chromosomes 4B and 7B, indicating that the detected shoot length QTLs are expressed under both optimum and heat-stressed growth conditions, thus they are not necessarily related to heat stress.

For heat response of shoot length, three QTLs were detected on chromosomes 3B and 7D (**Figure 4C** and **Supplementary Table S2**). On chromosome 3B, two QTLs (*QSLHR.nri-3B.1 and QSLHR.nri-3B.2*) were found, one mapped at 10 cM and the second one at 67 cM, together explaining 11.8% of the phenotypic variation in heat stress response of shoot length. The third QTL (*QSLHR.nri-7D*) was located at 27 cM on chromosome 7D. This QTL was represented by two SNPs, which collectively explained 12.8% of the phenotypic variation in heat stress response. In short, as the same QTLs were detected under optimal and heat-stressed growth conditions, shoot length QTLs responding to heat stress were only found by mapping heat stress response of the trait on chromosomes 3B and 7D (**Table 2**).

At optimal growth condition, four QTLs associated with the number of leaves per seedling were detected at genetic positions of 56, 77–78, 177–181, and 68–72 cM on chromosomes 1B, 2A, 3A, and 4B, respectively (**Figure 5A** and **Supplementary Table S2**). The SNP markers representing the QTLs explained 5.8–8.9% of total phenotypic variation in the number of leaves per seedling at the optimal growth condition. The two most significant SNP markers (IWB40186 and IWB25267) were co-localized at 78 cM on chromosome 2A, explaining 17.2% of the phenotypic variation in the number of leaves per seedling. The third most significant SNP was mapped at 68 cM on chromosome 4B, which accounted for 7.4% of phenotypic variation.

At heat-stressed growth condition, four QTLs significantly associated with number of leaves per seedling were detected (**Figure 5B** and **Supplementary Table S2**). The first QTL (*QLNHS.nri-1B*) was mapped at 112 cM on chromosome 3A, and explained about 6.2% of the phenotypic variation. The second (*QLNHS.nri-3B*), third (*QLNHS.nri-4B*), and fourth QTLs (*QLNHS.nri-5A*) were located at genetic positions of 66, 64, and 115 cM on chromosomes 3B, 4B, and 5A, respectively, and collectively explained 24.2% of the phenotypic variation in number of leaves per seedling at heat-stressed growth condition.

For heat stress response of number of leaves per seedling, seven QTLs, represented by 26 significant SNPs, were detected on chromosomes 2A, 3A, 4B, 5B, and 7B (**Figure 5C** and **Supplementary Table S2**). On chromosome 2A, two QTLs were found; one (*QLNHR.nri-2A.1*) mapped at 77–78 cM, and the other QTL (*QLNHR.nri-2A.2*) was located at 150 cM. The QTL (*QLNHR.nri-3A*) on 3A was located at 177 cM, while the one on 4B (*QLNHR.nri-4B*) was mapped at genetic position of 68–71 cM. Furthermore, two QTLs, *QLNHR.nri-5B.1* and *QLHR.nri-5B.2* were located at 49 and 144 cM, respectively, on chromosome 5A, while one QTL, *QLNHR.nri-7B* was found at 145 cM on chromosome 7B. The most significant SNP markers were IWB40186 and IWB25267, which were co-localized at 78 cM on chromosome 2A, and IWB61157, which was mapped at 150 cM on the same chromosome. The two markers mapped at 78 cM together explained 15.2% of the phenotypic variation, while the marker located at 150 cM accounted for 8.3% of the phenotypic variation in heat stress response of number of leaves per seedling. Overall, the data suggest that heat stress or heat response QTLs associated with the number of leaves per seedling are located on chromosomes 2A, 3A, 3B, 4B, 5A, 5B, and 7B according to QTLs detected for heat stress response of the trait, or by comparing the QTLs detected under heat-stressed vs. the optimum condition (**Table 2**).

For seedling recovery after removal of heat stress treatment, six QTLs were detected on chromosomes 2A, 2B, 2D, 3A, 7A, and 7B, and these were represented by 16 SNPs (**Figure 6** and **Supplementary Table S2**). The phenotypic variation explained by these SNPs varied from 6.5 to 8.5%. On chromosome 2A, one QTL (*QSLHS.nri-2A*) was located at genetic position of 96 cM. This QTL was represented by five SNPs, which collectively explained 33% of the phenotypic variation in seedling recovery after heat stress. The second QTL (*QSLHS.nri-2B*) was found on chromosome 2B at 19 cM, which accounted for 6.5% of the phenotypic variation. Another QTL (*QSLHS.nri-2D*) was found on chromosome 2D at the genetic distance of 26 cM, and it was represented by three SNP markers, which together explained 18.6% of the phenotypic variation. On chromosome 3A, one QTL (*QSLHS.nri-3A*) was detected and mapped at 123–129 cM. The QTL on 3A explained 12.4% of the phenotypic variation in seedling recovery after removal of heat stress treatment. In addition, one QTL (*QSLHS.nri-7A*) was identified on chromosome 7A at position 42–43 cM, while another one (*QSLHS.nri-7B*) was found at 90 cM on chromosome 7B. The QTLs on 7A and 7B accounted for 13.4 and 23.3% of the phenotypic variation in seedling recovery, respectively.

## Discussion

Wheat is one of the most important food and feed crops in the world. In the Southern Plains of the United States including Oklahoma and Texas where livestock and forage production are the largest contributors to agricultural income, winter wheat is often used for cool season grazing, which needs early planting for increased fall to winter forage production. Winter wheat under a dual-purpose management system could be planted as early as the end of August, when the temperature is often still very high for the crop to establish. Therefore, improving seedling heat tolerance for winter wheat grown for forage and grain production will have a huge economic impact in the region. We conducted a GWAS to map QTLs and identify SNP markers associated with seedling heat tolerance for MAS of seedling heat tolerance during wheat breeding. Identification of QTLs associated with seedling heat tolerance will facilitate the introgression of heat tolerance alleles into elite wheat cultivars through MAS.

In the present study, the association mapping panel showed significant phenotypic variation in leaf chlorophyll content, shoot length, number of leaves per seedling at optimal and high temperature regimes, and seedling recovery after removal of heat stress treatment. In addition, variation in heat stress response, i.e., relative performance difference between the two temperatures, for all traits was also observed. These results suggest that there is a great potential that these lines can be used to mine alleles for seedling heat tolerance for introgression into elite winter wheat lines for seedling heat tolerance improvement.

Population structure and familial relatedness can result in false positives in GWAS (Crossa et al., 2007; Matthies et al., 2012). Therefore, when GWAS is conducted, these parameters need to be considered in the model. In the present study, the level of genetic structure of the panel was assessed by the PCA, NJ tree, and STRUCTURE analyses. Results from the three clustering methods showed that this panel is structured into four major groups. Our results agree with previous GWAS done using winter wheat lines selected from the same hard red winter wheat association mapping panel (Ayana, 2017). In their study, they used 294 lines of the association mapping panel to molecular characterize spot blotch and bacterial leaf streak resistance in bread wheat, and the STRUCTURE analysis revealed four major groups existing in this panel, although admixtures were also observed. In the present study, the stratification was mainly based on geographical regions and pedigree relation. Genetic structuring of winter wheat lines along geographic regions has also been previously reported (Li et al., 2016; Liu et al., 2017).

In this study, we observed that some lines with common parents in their pedigrees tended to cluster in the same subgroup within the main group. For instance, some lines with “Jagger” wheat line as one of the parents in their pedigrees formed one subgroup. This result corroborates the GWAS of powdery mildew disease using a different set of winter wheat lines, in which the authors found that the lines were structured along pedigree information (Liu et al., 2017). Specifically, they found that 13 accessions with the common parent, “Jagger” in their pedigrees clustered in one group. However, in general, we observed that the level of genetic stratification was low, as revealed by the modest contribution of the three PCs (19.4%) to the total genetic variance. This reduced and loose population stratification is because of historical admixture resulting from sharing genetic materials among different wheat breeding programs in the hard red winter wheat region of the United States.

Linkage disequilibrium is one of the most important factors in association mapping studies because it determines the power of association between QTLs and phenotype. In this study, we estimated the LD decay distances of the three wheat genomes including A, B, and D genomes. Our results suggest that D genome had the highest LD decay distance (10.9 cM) compared to A (9.7 cM) and B (9.8 cM) genomes. As only 200 representative lines were selected from the original panel in the current study, the LD distances are changed compared to a previous study involving the same panel (Ayana, 2017). In general, our results corroborate previous studies done in wheat (Zhang et al., 2010; Hao et al., 2011; Ayana, 2017). However, other studies have reported much higher LD than estimated in our study when using different ecotype wheat lines. For example, LD decay distance of 23 cM in European hexaploid wheat lines has been reported (Nielsen et al., 2014).

In this study, three statistical models were compared to assess their ability to map QTLs and identify SNPs associated with seedling heat tolerance. We decided to do this because previous studies have shown that the best model can vary depending on the trait (Gurung et al., 2014). Finally, we selected the MLM, which accounts for both population structure (PCs) and K matrix, because of its statistical power to control false positives. To the best of our knowledge, rare QTL studies have been done for heat tolerance during the seedling stage of wheat. However, there have been a lot of QTLs studies in heat tolerance during the flowering stage or grain filling stage of wheat. For example, QTLs for heat tolerance during the grain filling stage of wheat have been reported (Vijayalakshmi et al., 2010; Talukder et al., 2014). Similarly, in other cereal crops such as sorghum (Chen et al., 2017; Chopra et al., 2017) and rice (Lafarge et al., 2017), QTL studies for heat tolerance have been conducted. The focus of previous studies was either on the vegetative stage or the flowering stage because heat stress during the flowering stage has been one of the most important limiting factors contributing to yield losses in many crop species. However, heat stress during the seedling stage of winter wheat has been a common issue in the southern Great Plains of the United States due to early planting, particularly in a dual-purpose management system, in which case the crop is planted very early in the fall. Therefore, this study was primarily conducted to unravel QTLs or genes associated with seedling heat tolerance in winter wheat purposely grown for forage as well as grain production.

Using the MLM, we identified multiple significant QTLs for wheat seedling traits at optimum and heat-stressed growth conditions. QTLs associated with seedling heat stress or heat response were found by comparing the QTLs detected under heat-stressed vs. the optimum condition, or mapping heat response QTLs using the relative phenotypic trait difference between the two growth conditions. QTLs associated with leaf chlorophyll content at heat-stressed growth condition but not at optimum temperature were found on chromosomes 2B, 2D, 4A, and 4B, while QTLs for heat stress response of the trait were detected on chromosomes 2B, 2D, 4A, 4B, and 5B (**Table 2**). We believe that these are the true chromosomes that harbor leaf chlorophyll content QTLs responding to heat stress since they were only detected under heat stressed temperature and/or mapped using heat response of the trait. Previous studies also identified QTLs for heat stress tolerance traits, specifically at grain filling stage of wheat on chromosomes 2B, 2D, and 4A (Paliwal et al., 2012; Acuña-Galindo et al., 2015; Bhusal et al., 2017). However, previous QTL studies conducted in wheat also identified QTLs for leaf chlorophyll content under heat stress on other chromosomes including 1B, 1D, 6A, and 7A (Talukder et al., 2014), which were not detected at the seedling stage in the current study. Moreover, in another QTL study, leaf chlorophyll content QTLs under heat stress mapped on chromosomes 1A and 6B were reported (Tahmasebi et al., 2016). Again, these QTLs were not detected in the present study. However, the QTL for heat-stress response of the leaf chlorophyll content detected on 5B was not reported in other studies mentioned above. Although QTLs detected at optimal temperature are not related to heat stress, interestingly in this study one SNP marker (IWB14950) on 1B, which associated with leaf chlorophyll content at optimal growth condition, has high sequence similarity with kDa class VI heat shock protein, known to be involved in heat stress tolerance. However, this SNP marker was not detected at heat-stressed growth condition as well as heat stress response.

We also conducted BLAST search on NCBI to unravel candidate genes using sequences of the SNPs detected in the present study (**Supplementary Table S2**). The results showed that some of the significant SNP markers have high sequence similarities with candidate genes, known to be involved in plant stress responses in different crops including wheat. For example, on chromosome 2D, the significant SNP IWB28728 for leaf chlorophyll content responding to heat stress has 89% sequence similarity with putative plastid-lipid-associated protein 13. The putative plastid lipid-associated protein 13 has been reported to play an important role in improving plant performance under stress conditions. In addition, it actively participates in thylakoid function from biogenesis to senescence, suggesting that it is a precursor of the chloroplast thylakoid membranes (Rottet et al., 2015). Similarly, on chromosome 4B, significant SNP IWB42264 for leaf chlorophyll content at heat-stressed growth condition and heat response of the trait, has 94% sequence similarity with K (+) efflux antiporter 5 isoform X1, which contains potassium (K^+^), a major osmoticum of plant cells. The accumulation of potassium (K^+^) in the plant vacuole is important for plants under high-salt stressed conditions (Assaha et al., 2017). In addition, the significant SNP IWB18745 for heat stress response of leaf chlorophyll content on chromosome 2D has 97% sequence similarity with IAA-amino acid hydrolase ILR1-like, which is able to hydrolyze certain amino acid conjugates of the plant growth regulator indole-3-acetic acid (IAA) (LeClere et al., 2002). Moreover, on chromosome 2D, the heat responding SNP IWB4541 has a DNA sequence with 100% similarity to that of the heat shock N-terminal domain-containing protein found in maize, which is essentially involved in plant responses to various environmental stress including heat.

For shoot length, the same significant QTLs were detected at both optimal and heat-stressed growth conditions. Generally, QTLs associated with a trait under optimal conditions usually controls the trait under stressed-conditions (Mathews et al., 2008; Mwadzingeni et al., 2017). In the present study, this scenario was observed for shoot length QTLs on chromosomes 4B and 7B indicating that the detected QTLs were associated with shoot length itself as a plant architecture trait, and not related with heat stress tolerance *per se*. These results suggest that the effects of these QTLs are not influenced by temperature changes. Therefore, such kind of QTLs may be useful in marker-assisted breeding (MAB) of crops with broad environmental adaptation. On the other hand, shoot length QTLs were detected for heat response on 3B and 7D, which were also reported previously to harbor QTLs for heat tolerance traits at vegetative and grain filling stages of wheat (Vijayalakshmi et al., 2010; Paliwal et al., 2012).

Although some of the markers associated with shoot length were significant at both growth conditions, BLAST search revealed that some of the identified SNPs have high sequence similarities with candidate genes known for plant stress response. For example, the DNA sequence of SNP IWB35611 on chromosome 4B has high sequence similarity with serine/threonine protein kinase STE 20-like, which has been reported to play an important role in salt tolerance in plants (Liang et al., 2011). Another SNP IWB12856 on chromosome 4B has high sequence similarity with inositol-tetrakisphosphate 1-kinase 3, transcript variant X1, which has been reported to confer plant stress tolerance (Yang et al., 2008). The two SNP markers on 4B were located 2.45 cM apart from each other. In addition, the SNP marker IWB1428 on 3B, which was found to be significantly associated with heat stress response of shoot length, showed 83% sequence similarity with G-type lectin S-receptor-like serine/threonine protein kinase. Research done in the past showed that the G-type lectin S-receptor-like serine/threonine protein kinase acts as a positive regulator of plant tolerance to salt stress (Deng et al., 2009; Sun et al., 2013).

Similarly, for the number of leaves per seedling and seedling recovery, some of the QTLs detected in this study were located in the same chromosomes that were reported in other heat stress studies at various adult plant stages (Mason et al., 2010; Vijayalakshmi et al., 2010; Paliwal et al., 2012; Talukder et al., 2014; Acuña-Galindo et al., 2015). However, BLAST search against sequences of SNPs associated with the number of leaves per seedling and seedling recovery did not reveal any candidate genes that are known responding to abiotic stress.

In summary, some QTLs for seedling heat tolerance-related traits identified in this study were found on the same chromosomes previously reported to harbor QTLs for heat tolerance, although the growth stages reported in the previous studies are different from the growth stage investigated in the present study. Our results suggest that some of heat tolerance QTLs detected during the seedling and the flowering stages of wheat may be co-localized. In addition, other QTLs identified in the seedling stage in the present study have not been reported in those studies conducted at the flowering time or grain filling stages. Moreover, BLAST search using DNA sequences of some of the significant loci found in this study revealed candidate genes known to be involved in plant stress responses in wheat and other crop species. To the best of our knowledge, this is the first GWAS to map QTLs and identify SNP markers significantly associated with seedling heat tolerance-related traits targeting early planting of dual-purpose winter wheat. Significant SNP markers identified in this study will be used for MAS of seedling heat tolerance to facilitate selection of the trait during wheat breeding.

## Author Contributions

FM phenotyped the association mapping panel, analyzed both phenotypic and genotypic data, and drafted the manuscript. HA helped in the candidate gene search and review of the manuscript. JA assisted in experiment implementation and review of the manuscript. TK helped in data collection and review of the manuscript. WH helped in review of manuscript. X-FM supervised the study and finalized the manuscript. All authors read and approved the manuscript.

## Conflict of Interest Statement

The authors declare that the research was conducted in the absence of any commercial or financial relationships that could be construed as a potential conflict of interest.

## Footnotes

1 http://www.triticeaecap.org

2 https://urgi.versailles.inra.fr/

## Supplementary Material

FIGURE S1 Structure analysis of the 200 winter wheat lines from the association mapping panel. **(A)** Neighbor-joining (NJ) tree; **(B)** population structure.

FIGURE S2 Scatter plots showing the linkage disequilibrium (LD) decay curves of the three subgenomes estimated in the association mapping panel. The LD estimates (*r*^2^) for pairs of SNP markers were plotted against the genetic distance in cM.

FIGURE S3 The quantile-quantile (Q-Q) plots of the mixed liner model applied to the investigated traits. **(A–C)** Leaf chlorophyll content at optimum temperature, heat-stressed growth condition, and heat response of the trait; **(D–F)** shoot length (cm) at optimum temperature, heat-stressed growth condition, and heat response of the trait; **(G-I)** Number of leaves at optimum temperature, heat-stressed growth condition, and heat response of the trait; **(J)** Seedling recovery after removal of the heat stress.

TABLE S1 Linkage disequilibrium analysis of 200 lines in the hard red winter association mapping panel.

TABLE S2 The QTL and the significant SNP markers associated with seedling traits of wheat at optimal and heat-stressed growth conditions.
