# Supplementary figures and images for: Genome-Wide Association Mapping of Seedling Heat Tolerance in Winter Wheat
Source: Front Plant Sci. 2018 Sep 4;9:1272. doi: 10.3389/fpls.2018.01272 (PMC6131858; doi:10.3389/fpls.2018.01272)

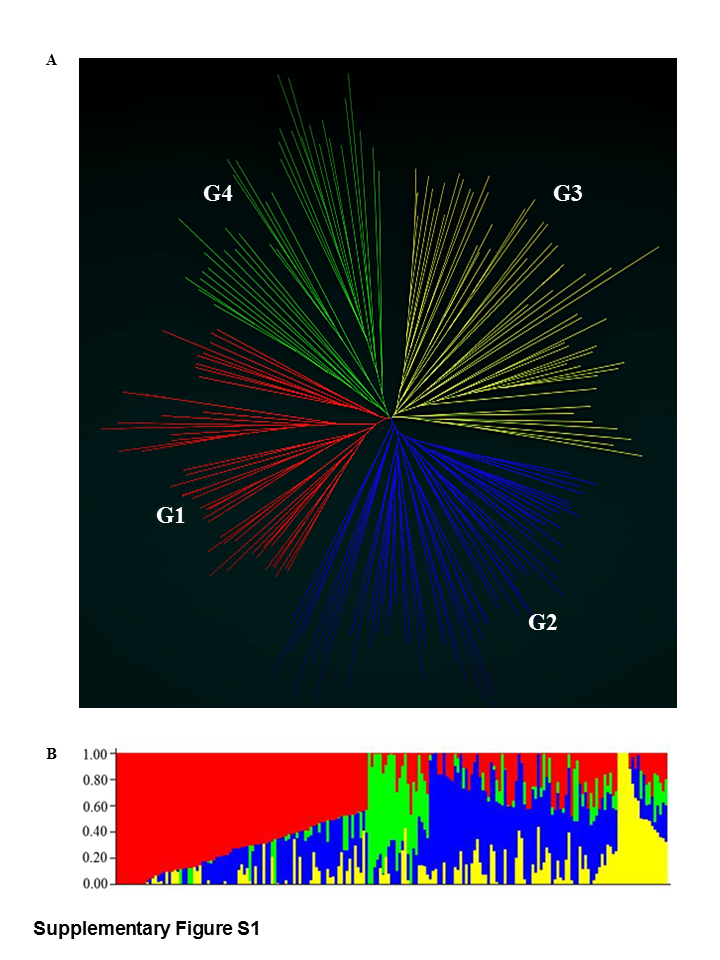

Supplement: FIGURE S1 — Structure analysis of the 200 winter wheat lines from the association mapping panel. (A) Neighbor-joining (NJ) tree; (B) population structure. [file Image_1.TIF]

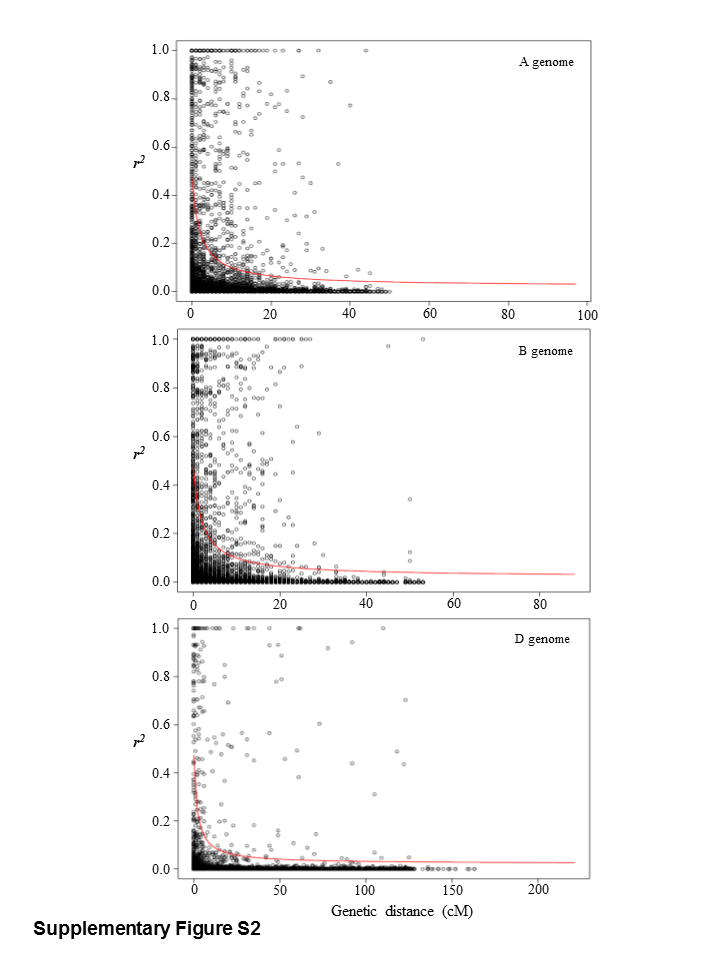

Supplement: FIGURE S2 — Scatter plots showing the linkage disequilibrium (LD) decay curves of the three subgenomes estimated in the association mapping panel. The LD estimates (r2) for pairs of SNP markers were plotted against the genetic distance in cM. [file Image_2.TIF]

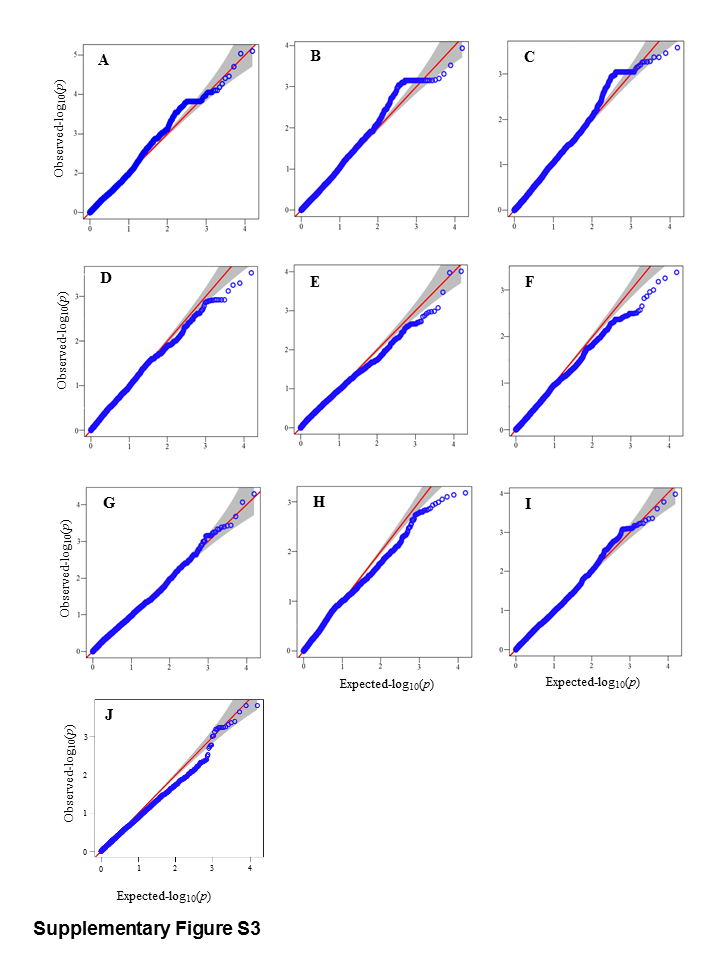

Supplement: FIGURE S3 — The quantile-quantile (Q-Q) plots of the mixed liner model applied to the investigated traits. (A–C) Leaf chlorophyll content at optimum temperature, heat-stressed growth condition, and heat response of the trait; (D–F) shoot length (cm) at optimum temperature, heat-stressed growth condition, and heat response of the trait; (G-I) Number of leaves at optimum temperature, heat-stressed growth condition, and heat response of the trait; (J) Seedling recovery after removal of the heat stress. [file Image_3.TIF]
